# Oxidative stability and emission profiles of biodiesel from hemp, karanja, castor, and amla oils: impact of natural antioxidants

**DOI:** 10.1039/d5ra05704c

**Published:** 2025-10-20

**Authors:** Humaira Kanwal, Farooq Anwar, Ahsan Tanvir, Abu Bakar Siddique, Sobia Tariq, Sidra Aslam, Saiqa Yaqoob

**Affiliations:** a Institute of Chemistry, University of Sargodha Sargodha 40100 Pakistan humachaudhary48@yahoo.com abubakar.siddique@uos.edu.pk; b Applied Chemistry Research Center, Pakistan Council of Scientific and Industrial Research Laboratories Complex Ferozepur Road Lahore 54600 Pakistan; c Department of Food Science, Faculty of Food Science & Technology, Universiti Putra Malaysia 43400 Serdang Selangor Malaysia; d Institute of Carbon Neutral Technology, Shenzhen Polytechnic University China; e College of Pharmacy, University of Sargodha Sargodha 40100 Pakistan; f Department of Engineering & Informatics, Technological University of Shannon Midlands Midwest Athlone Campus Ireland

## Abstract

Biodiesel is a renewable fuel with great potential, but its quality can decline over time due to oxidation, which forms harmful compounds like peroxides, aldehydes, and alcohols. This study examines the oxidative stability and storage life of biodiesel made from non-edible oils such as hemp, karanja, castor, and amla. The stability was evaluated using parameters, like peroxide value, totox value, conjugated diene and triene values, and *para*-anisidine value. To improve stability, both synthetic and natural antioxidants were tested. Butylated hydroxyanisole (BHA) was used as a synthetic antioxidant, while natural antioxidants used were aqueous extract of *Moringa oleifera* leaves, citrus fruit residues, and olive pomace. BHA provided the best overall protection, and among the natural antioxidants, olive pomace extract performed the best. Engine emission tests were also carried out to study the environmental impact of these additives. The results showed that *Moringa oleifera* extract reduced the high NO_*x*_ emissions commonly seen with biodiesel, while all antioxidants caused only a slight increase in CO and unburned hydrocarbons. Overall, the emissions from biodiesel remained lower than those from conventional diesel fuel.

## Abstract 2

Effect of natural and synthetic antioxidants on the oxidative stability and emission profiling of combustion of hemp, karanja, castor, and amla oil-based biodiesels.

## Graphical abstract

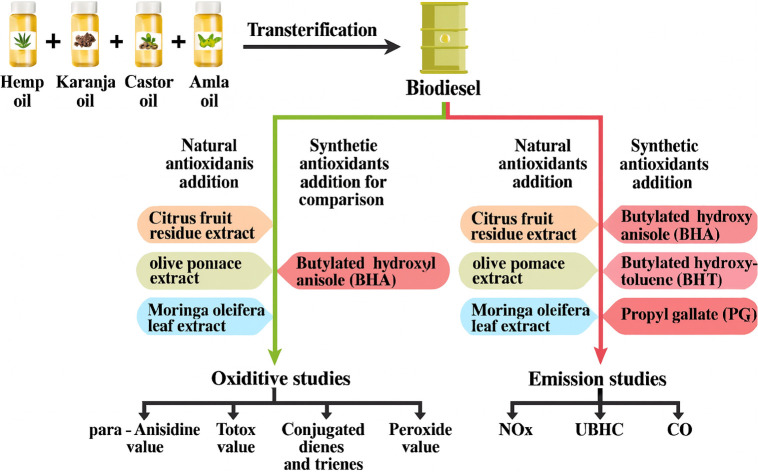

## 1 Introduction

Biodiesel, a renewable and biodegradable fuel derived from vegetable oils and animal fats, has gained significant attention as a sustainable alternative to conventional petro-diesel. It mainly consists of fatty acid methyl esters (FAMEs) produced through transesterification of triglycerides with short-chain alcohols in the presence of a catalyst.^1,2^ Plant-derived triglycerides typically have a high proportion of unsaturated fatty acids, making the resulting biodiesels more prone to oxidative degradation.^3^ According to Zambiazi *et al.* (2007), although some oils such as rice, peanut, cotton, and corn contain relatively higher levels of saturated fatty acids, their overall composition remains dominated by unsaturated ones, with saturated content generally below 40%.^4^

The predominance of unsaturated esters makes biodiesel susceptible to oxidation under normal storage and operating conditions. During oxidation, reactive free radicals attack double bonds in unsaturated fatty acids, forming hydroperoxides that subsequently decompose into secondary oxidation products such as aldehydes, ketones, and alcohols.^5–7^ These reactions cause undesirable changes in fuel properties, including increased viscosity, acidity, and gum formation which impair combustion efficiency and storage stability. For instance, Suraj *et al.* (2021) observed that after one year of storage, Karanja biodiesel showed an 11-fold increase in peroxide value and a threefold rise in acid value, accompanied by increased viscosity and surface tension, adversely affecting injection performance.^8^

Despite its environmental advantages, oxidative instability and poor cold flow behavior remain major challenges that hinder the large-scale commercialization of biodiesel.^9^ Various strategies have been explored to counter oxidation, including structural modification,^10^ blending with diesel or mixed feedstocks,^11,12^ and the addition of antioxidants.^9,13^ Among these, the use of antioxidants has proven to be one of the most effective and practical approaches. Antioxidants inhibit free radicals and interrupt the chain reaction of autoxidation, thereby prolonging biodiesel shelf life.^7,14,15^ Synthetic antioxidants such as pyrogallol (PY), tert-butylhydroquinone (TBHQ), butylated hydroxyanisole (BHA), propyl gallate (PG), and butylated hydroxytoluene (BHT) have been widely applied due to their high efficiency.^14,16^ However, their environmental persistence, toxicity, and potential bioaccumulation in ecosystems have raised increasing concerns.^17^

Research gaps still exist in this field. Firstly, while synthetic antioxidants have been extensively studied, comparative data on natural antioxidants derived from plant sources remain limited, especially concerning their dual influence on oxidative stability and emission characteristics. Secondly, most previous studies have focused on a single feedstock, with little information available on non-edible oil-based biodiesels such as those from *Cannabis sativa* L. Thirdly, although antioxidants are known to improve storage stability, their effects on engine exhaust emissions particularly NO_*x*_, CO, and unburned hydrocarbons (UBHC) have not been comprehensively evaluated.^18–20^

To address these gaps, the present study investigates the impact of both natural and synthetic antioxidants on the oxidative stability and emission characteristics of biodiesel produced from four non-edible oil sources: *Cannabis sativa* L., *Pongamia pinnata* L., *Ricinus communis* L., and *Phyllanthus emblica* L. Natural antioxidants including *Moringa oleifera* leaf extract, citrus fruit residue extract, and olive pomace extract—were evaluated due to their appreciable antioxidant potential reported in the literature,^21–23^ alongside the synthetic antioxidant BHA as a reference. Additionally, PG and BHT were incorporated for emission studies. The oxidative stability was assessed using peroxide, totox, conjugated diene and triene, and *para*-anisidine values, while the emission performance was evaluated by measuring CO, UBHC, and NO_*x*_ emissions in a diesel engine. This comprehensive approach aims to establish a comparative understanding of how natural antioxidants can enhance both the fuel stability and environmental sustainability of biodiesel, providing valuable insights for green fuel development.

## 2 Experimental work

### 2.1 Material and methods

All chemicals and reagents used were of analytical grade to ensure accuracy and reproducibility in experimental analyses. Non-edible oils extracted from *Cannabis sativa* L. (hemp), *Pongamia pinnata* L. (karanja), *Ricinus communis* L. (castor), and *Phyllanthus emblica* L. (amla) were used as feed stocks for biodiesel production. The biodiesels were synthesized through a base-catalyzed transesterification process using analytical-grade methanol (CH_3_OH) and potassium hydroxide (KOH) as the alcohol and catalyst, respectively.^24^ The reaction was carried out in a three-necked round-bottom flask equipped with a reflux condenser, magnetic stirrer, and thermometer. The molar ratio of methanol to oil was maintained at 6 : 1, with 1% (w/w) KOH as the catalyst. The mixture was stirred continuously at 60 ± 2 °C for 90 minutes. After completion, the reaction mixture was allowed to settle for 8 hours to enable phase separation. The lower glycerol layer was removed, and the upper biodiesel layer was washed with warm distilled water to remove residual catalyst and soap, followed by drying at 105 °C. The purified biodiesels were stored in airtight amber bottles at room temperature for further analysis.

### 2.2 Improvement in oxidative stability of biodiesels with antioxidants

In order to assess the effect of synthetic and natural antioxidant additives on the oxidative stability of biodiesels, selected biodiesels: Hemp oil methyl esters (HOMEs), Karanja oil methyl esters (KOMEs), Castor oil methyl esters (COMEs) and Amla oil methyl esters (AOMEs) were stabilized with three natural antioxidant extracts including citrus fruit residue extract, olive pomace extract and *Moringa oleifera* leaf extract while a synthetic antioxidant compound *i.e.* BHA was used as a positive control for comparison purpose. The crude concentrated natural antioxidant extracts were prepared by extracting the bioactives of selected plants' dried material with 80% alcohol (on orbital shaker) followed by filtration and concentration of extract using a rotary evaporator.^25^

The prepared extracts, previously evaluated for their antioxidant potential through TPC, TFC, and DPPH radical scavenging assays,^21,23,26^ along with BHA (used as a positive control), were incorporated into pre-heated biodiesel samples at a concentration of 200 ppm (w/w). The mixtures were stirred for 30 minutes at 50 °C to ensure uniform dispersion. The stabilized biodiesel samples, along with the control (without any antioxidant or additive), were stored under ambient conditions for a period of four weeks. During the storage period, samples were analyzed weekly to monitor oxidative degradation by determining the peroxide value (PV), *p*-anisidine value (P-AV), totox value, and the levels of conjugated dienes and trienes.

Biodiesel oxidation is a complex, multi-step process, and no single analytical test can fully characterize its progression. The PV reflects the extent of primary oxidation by quantifying hydroperoxide formation at the initial stages. The P-AV measures secondary oxidation products, primarily aldehydes, indicating fuel degradation over time. The formation of conjugated dienes and trienes provides insight into structural alterations in unsaturated fatty acid chains that may promote polymerization and sediment formation. The totox value offers an integrated measure of total oxidation, combining information from both primary and secondary oxidation stages.

By employing these complementary assays, a comprehensive oxidative stability profile of the biodiesel samples was established, enabling precise evaluation of the extent and progression of oxidation, as well as the comparative effectiveness of natural and synthetic antioxidants. The oxidative stability of biodiesel was assessed using the aforementioned parameters, determined in accordance with standard IUPAC methods.^27^

### 2.3 Peroxide value (PV)

PV mainly represents the extent of primary oxidation (peroxides and hydroperoxides formed due to oxidation). PV of stabilized and control biodiesels was determined according to IUPAC method.^27^ Briefly, approximately 2 g of biodiesel sample was dissolved in a freshly prepared acetic acid–chloroform mixture (3 : 2, v/v) to obtain a clear solution. To this, 0.5 mL of saturated potassium iodide (KI) solution was added, and the mixture was kept in the dark for 1 min with occasional shaking to liberate iodine. Subsequently, 30 mL of distilled water was added, and the liberated iodine was titrated against standardized 0.01 N sodium thiosulfate solution using starch as an indicator until the blue color disappeared. A reagent blank was performed under identical conditions. The peroxide value, expressed as milliequivalents of active oxygen per kilogram of sample (meq. O_2_ per kg), was calculated using the standard procedure based on the titration volumes and sample mass.

### 2.4 *Para*-anisidine value (P-AV)

P-AV measures the extent of secondary oxidation [(mainly unsaturated aldehydes (2, 4-dienals & 2-alkenals))] and was determined *via* IUPAC protocol.^27^ For this test, 5 g of biodiesel sample was placed in the volumetric flask and diluted with *iso*-octane. After this, two test tubes were taken, one of which having 5 mL of test solution (biodiesel + solvent) while in other blank (solvent) was placed. Then *para*-anisidine reagent was added to both test tubes and mixed well. Absorbance of both the sets (blank and sample solution/reaction mixture) was noted at 350 nm using UV-Vis spectrophotometer (U-2001, model 121-0032, Hitachi Instruments Inc., Tokyo, Japan). P-AV was determined using eqn (1).1
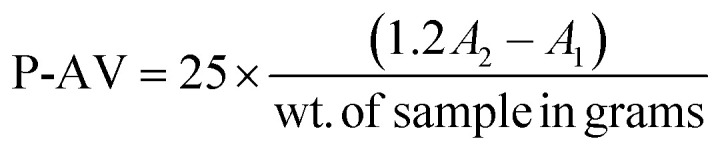
where in *A*_1_ and *A*_2_ presents the absorbance of the fat solution in *iso*-octane before and after reaction with *para*-anisidine, respectively.

### 2.5 Conjugated dienes and trienes

Conjugated dienes and conjugated trienes values were measured using the standard procedure reported in the literature.^28^ Briefly, 0.01 g of biodiesel sample oil was taken and diluted with *iso*-octane. Corresponding to CD and CT, absorbance of stabilized biodiesel and control was taken at 232 nm and 268 nm using spectrophotometer (U-2001, model 121-0032, Hitachi Instruments Inc., Tokyo, Japan).

### 2.6 Totox value

The totox value is known as the sum of 2 PV and P-AV and was calculated using the data of PV and *p*-anisidine values.^27^

### 2.7 Exhaust emission studies

The emission characteristics of the synthesized biodiesel samples were evaluated using commercial petro-diesel as a reference fuel.^29^ In addition to pure biodiesel (B100) and its blends with petro-diesel, biodiesel samples containing both natural and synthetic antioxidants were tested to assess the influence of these additives on exhaust emission behavior. The natural antioxidants employed in this study included *Moringa oleifera* extract, olive pomace extract, and citrus fruit residue extract, while the synthetic antioxidants comprised of BHA, BHT, and PG. Prior to each experimental run, the diesel engine was pre-heated for 10–15 minutes under optimum operating conditions to ensure temperature stabilization and consistent combustion performance. Each test was performed in triplicate to minimize experimental error and enhance data reliability. The biodiesel blends used for emission testing included B5 (5% biodiesel + 95% petro-diesel), B20 (20% biodiesel + 80% petro-diesel), B40 (40% biodiesel + 60% petro-diesel), B60 (60% biodiesel + 40% petro-diesel), and B100 (100% biodiesel), along with B20 formulations supplemented with the respective antioxidants or additives. All samples were combusted at full engine load to enable comparative assessment of their emission profiles under standardized conditions.

The KAM-SD-1100B engine model was used for emission studies; the engine stroke was 125 mm, power was 11.75 kW, and speed was 2200 rpm while minimum fuel consumption was 2.5 L h^−1^. CO, NO_*x*_ and UBHC emissions from the biodiesel blends and the effects of additives on the emissions were estimated by the help of Testo 350-XL fuel gas analyzer (Testo.454 version. U.S.). The emission results were evaluated by statistical software (*i.e.*, MS Excel and SSPS, and MINITAB).

## 3 Results and discussions

### 3.1 Stability studies of biodiesels

Without antioxidants, biodiesel lacked oxidative stability due to development of primary and secondary oxidation products. In order to study the effect of antioxidants on the shelf-life of biodiesels, samples of HOMEs, KOMEs, COMEs and AOMEs were spiked/stabilized with known concentration (200 ppm) of BHA (synthetic antioxidant) and natural antioxidant extracts followed by storage for 28 days under ambient conditions. Typically, three natural antioxidant extracts including *Moringa oleifera* extract, olive pomace extract, and citrus fruit residue extract and BHA were used to stabilize the biodiesel against oxidative degradation. Antioxidant efficiency of the above said additives was measured by determining the PV, P-AV, totox value, and conjugated dienes and trienes values of the stabilized biodiesels. The selected oxidation parameters in the present research are known to be the important indictors towards assessment of oxidative deterioration of any oil or fat products.^25,27^ A characteristic trend in the increase of various oxidation parameters was observed across all biodiesel treatments. However, the stabilized biodiesels (HOMEs, KOMEs, COMEs, and AOMEs) exhibited a significantly lower rise in oxidative degradation indicators PV, P-AV, totox value, conjugated dienes, and conjugated trienes compared to the control sample (biodiesel without any antioxidant additive), as presented in Table 1. Among all treatments, biodiesels stabilized with BHA demonstrated the least oxidative deterioration, highlighting its superior antioxidant efficiency. Despite the effectiveness of synthetic antioxidants such as BHA, their application raises health and environmental concerns, particularly in contexts involving edible oils and food-grade products.^30,31^

**Table 1:** Relative change in peroxide, *p*-anisidine, conjugated diene, totox and conjugated triene value of hemp, karanja, castors and amla biodiesel after 28 days' storage^a^

Sample	Peroxide value (meq. per kg)	*p*-Anisidine value	Conjugated dienes content [1% *ε*_1_ cm (232 nm)]	Conjugated trienes [1% *ε*_1_ cm (268 nm)]	Totox value
HOMEs – Controlled	13.65	2.45	1.56	1.08	28.85
HOMEs-BHA	10.65	1.55	1.44	0.54	22.85
HOMEs-CE	12.00	2.25	1.39	0.95	26.25
HOMEs-ME	12.70	2.26	1.36	0.69	27.66
HOMEs-OE	11.65	2.37	1.53	0.89	25.65
KOMEs – Controlled	7.81	2.65	1.75	1.12	15.22
KOMEs-BHA	6.3	1.75	1.04	0.44	12.2
KOMEs-CE	7.29	2.45	1.07	0.62	13.7
KOMEs-ME	5.07	2.46	1.46	0.61	9.5
KOMEs-OE	4.15	2.57	1.41	0.71	7.23
COMEs – Controlled	13.55	2.47	1.56	1.23	29.574
COMEs-BHA	11.18	1.57	1.44	0.69	23.932
COMEs-CE	12.03	2.27	1.39	1.1	26.331
COMEs-ME	12.65	2.35	1.36	0.84	27.659
COMEs-OE	11.32	2.37	1.53	1.04	25.012
AOMEs – Controlled	13.9	2.7	1.81	1.48	29.1
AOMEs-BHA	10.9	1.8	1.69	0.94	23.1
AOMEs-CE	12.25	2.5	1.64	1.35	26.5
AOMEs-ME	12.95	2.51	1.61	1.09	27.91
AOMEs-OE	11.9	2.62	1.78	1.29	25.9

a HOMEs: hemp oil methyl esters, KOMEs: karanja oil methyl esters, COMEs: castor oil methyl esters, AOMEs: amla oil methyl esters, BHA: butylated hydroxyl anisole stabilized biodiesel, CE: stabilizes with citrus fruit residue extract, ME: stabilizes with *M. oleifera* leave extract, OE: stabilizes with olive pomace extract.

Among the natural antioxidants tested, *Moringa oleifera* extract and olive pomace extract emerged as the most potent stabilizers. COMEs mixed with *Moringa oleifera* extract (COMEs-ME) exhibited the most effective suppression of conjugated dienes (+1.36) and conjugated trienes (+0.84), demonstrating excellent control over lipid oxidation. Similarly, COMEs mixed with olive pomace extract (COMEs-OE) achieved the lowest increases in totox value (+25.01) and PV (+11.32 meq. per kg), indicating enhanced overall oxidative stability. In the case of HOMEs, stabilization with olive pomace extract resulted in relatively low changes in PV (+11.65 meq. per kg) and totox values (+25.65), further confirming olive pomace extract's strong ant oxidative potential.

For AOMEs, formulations containing citrus fruit extract and olive pomace extract were particularly effective in reducing P-AV (+2.5) and PV (+11.90 meq. per kg), respectively, while AOMEs mixed with *Moringa oleifera* extract (AOMEs-ME) exhibited notable control over conjugated diene and triene formation. KOME mixed olive pomace extract (KOME-OE) displayed the strongest anti oxidative performance overall, showing the lowest PV and totox values among all biodiesel types, thereby underscoring the high oxidative stability of karanja biodiesel.

Overall, among all natural antioxidants evaluated, olive pomace extract demonstrated the best performance by controlling the PV and totox values, *Moringa oleifera* extract was most effective in controlling conjugated dienes and trienes, and citrus fruit residue extract showed superior activity in reducing P-AV. The antioxidant efficiency of these natural extracts can be attributed to their rich composition of phenolic compounds inherent to *Moringa oleifera* leaves, citrus fruit residues, and olive pomace^23,32,33^ The present findings are consistent with previous studies reporting substantial improvements in the oxidative stability of biodiesels upon stabilization with plant-derived antioxidant extracts, reaffirming the potential of natural phenolic compounds as sustainable alternatives to synthetic additives.^34^

### 3.2 Exhaust emission studies of biodiesels

Climate change has emerged as one of the most critical environmental challenges of the modern era, with global warming recognized as a pressing climate emergency. Mitigating greenhouse gas emissions is therefore an urgent global priority. According to the EIA Energy Outlook (2020),^35^ coal consumption has declined sharply, while the adoption of renewable fuels has increased worldwide. Among these, biodiesel has gained considerable attention as a renewable, biodegradable, and cleaner burning alternative to fossil diesel, and its global demand is expected to continue rising in the coming years.^29,36^

Biodiesel combustion generally produces fewer hazardous emissions owing to its high oxygen content, resulting in reduced carbon monoxide (CO) and unburnt hydrocarbons (UBHCs) compared to conventional petro-diesel. However, the increased oxygen availability often leads to higher nitrogen oxides (NO_*x*_) emissions, one of the key challenges associated with biodiesel utilization. A practical strategy to mitigate NO_*x*_ emissions involves the incorporation of small quantities of antioxidants into biodiesel blends. Synthetic antioxidants such as PG, BHA, and BHT have demonstrated the ability to lower NO_*x*_ emissions by reducing oxygen availability during combustion, albeit with a slight trade-off in the form of increased CO and UBHC emissions. Moreover, many plant-based extracts possess inherent antioxidant activity and can similarly reduce NO_*x*_ emissions due to their high phenolic content. Consequently, in this study, cost-effective and locally available natural antioxidants were employed to develop an environmentally benign, *i.e.*, green fuel with performance characteristics comparable to conventional petro-diesel.

**Fig. 1:**
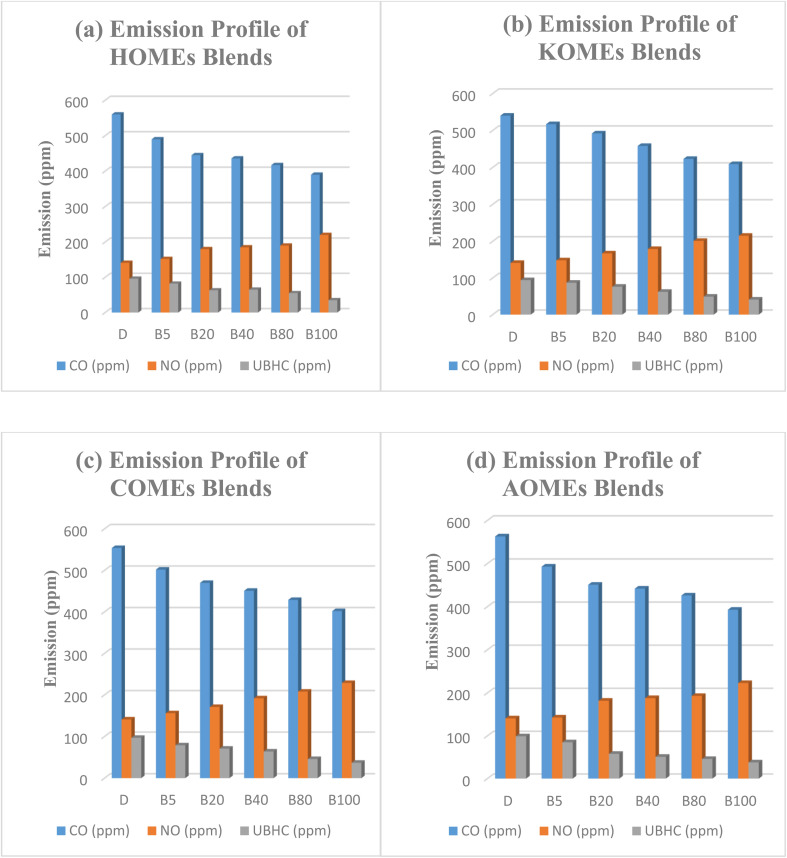
Emission profiles of petro-diesel blends of (a) HOMEs, (b) KOMEs, (c) COMEs and (d) AOMEs.

Emission studies were carried out using biodiesel–diesel blends of varying compositions, including B5 (5% biodiesel + 95% petro-diesel), B20 (20% biodiesel + 80% petro-diesel), B40 (40% biodiesel + 60% petro-diesel), B60 (60% biodiesel + 40% petro-diesel), and B100 (pure biodiesel). Subsequently, B20 blends of each biodiesel were fortified with different concentrations of three synthetic antioxidants (PG, BHA, BHT) and three plant-derived antioxidant extracts (*Moringa oleifera*, olive pomace, and citrus fruit residues). The emission profiles of all fuel samples are presented in Fig. 1, with pure petro-diesel (D) included for reference.

Comparative analysis of the D, B5, B20, B40, B60, and B100 blends revealed that petro-diesel combustion produced higher CO and UBHC emissions than biodiesel blends. As the biodiesel fraction increased, CO and UBHC emissions decreased, whereas NO_*x*_ emissions showed a proportional increase. This emission behavior was consistent across all tested biodiesels. Specifically, B20 blends of hemp, karanja, castor, and amla biodiesels exhibited 25%, 18%, 21%, and 29% higher NO_*x*_ emissions, respectively, compared to petro-diesel. These findings align with previous studies reporting that NO_*x*_ generation is influenced by combustion chamber temperature, fuel oxygen concentration, and enthalpy of vaporization.^37,38^ The elevated NO_*x*_ emissions observed with higher biodiesel content can thus be attributed to the increased oxygen availability in biodiesel, which promotes more complete combustion resulting in lower CO and UBHC emissions but higher flame temperatures, ultimately enhancing thermal NO_*x*_ formation.^38,39^

**Fig. 2:**
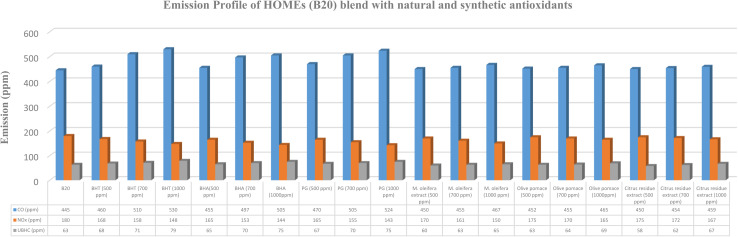
Emission profile of HOMEs (B20) blend with natural and synthetic antioxidants.

**Fig. 3:**
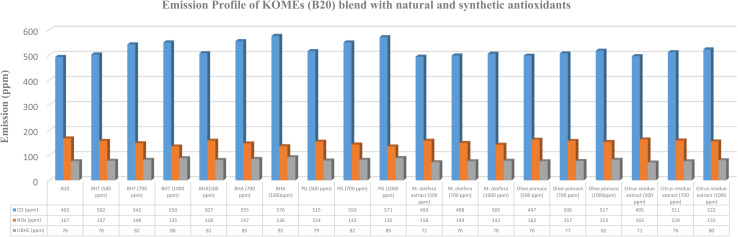
Emission profile of KOMEs (B20) blend with natural and synthetic antioxidants.

**Fig. 4:**
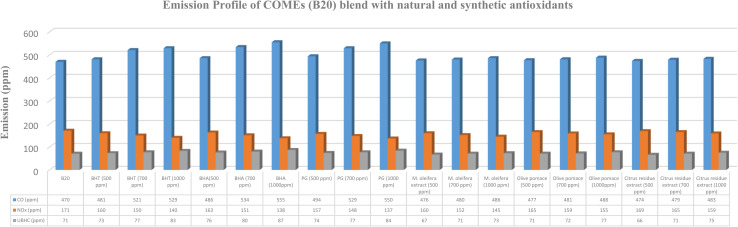
Emission profile of COMEs (B20) blend with natural and synthetic antioxidants.

**Fig. 5:**
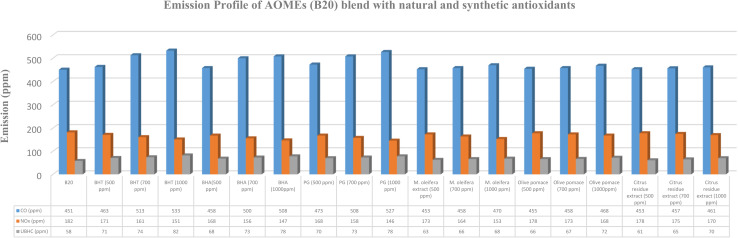
Emission profile of AOMEs (B20) blend with natural and synthetic antioxidants.

The influence of varying concentrations of synthetic and natural antioxidants on the emission characteristics of B20 biodiesel blends is illustrated in Fig. 2–5. The emission analysis revealed a distinct reduction in NO_*x*_ emissions upon the addition of synthetic antioxidants, with the extent of reduction increasing proportionally with antioxidant concentration. The maximum NO_*x*_ reduction (20.56%) was observed for B20 blends of HOMEs containing 1000 ppm of PG, followed by COMEs (19.91%), AOMEs (19.79%), and KOMEs (19.16%). BHA and BHT exhibited comparable trends, though BHA performed slightly better in several cases. Specifically, BHA reduced NO_*x*_ by 20.00% in HOMEs, 18.57% in KOMEs, 19.30% in COMEs, and 19.23% in AOMEs, while BHT achieved reductions of 17.78%, 19.16%, 18.14%, and 17.03%, respectively.

Although the inclusion of synthetic antioxidants effectively mitigated NO_*x*_ emissions, a concurrent increase in CO and UBHC emissions was observed. Among the synthetic antioxidants, BHA caused the smallest rise in CO emissions, with increases of 13.48%, 16.94%, 18.09%, and 12.63% for HOMEs, KOMEs, COMEs, and AOMEs, respectively, at 1000 ppm. For UBHC emissions, BHA again demonstrated the most favorable performance, showing increases of 19.05% in HOMEs, 21.05% in KOMEs, 22.54% in COMEs, and 34.48% in AOMEs. In comparison, BHT at 1000 ppm caused higher CO emissions (19.73% in HOMEs, 11.73% in KOMEs, 12.55% in COMEs, and 18.17% in AOMEs) and substantially greater increases in UBHCs, particularly in HOMEs (30.16%) and AOMEs (41.38%). PG at 1000 ppm produced moderate rises in CO and UBHC emissions, averaging 17.75% and 21.75%, respectively, across all biodiesels. Overall, BHA was identified as the most effective synthetic antioxidant in minimizing CO and UBHC increases while achieving considerable NO_*x*_ reduction. Importantly, CO emissions from all biodiesel blends, except karanja-based biodiesel, remained lower than those of petro-diesel. It is suggested that optimizing antioxidant concentration or selecting a milder antioxidant for karanja biodiesel may alleviate this issue. Collectively, the findings indicate that the synthetic antioxidants exhibit NO_*x*_ reduction efficiency in the order of PG > BHA > BHT, accompanied by CO and UBHC increases following the trend BHA < PG < BHT.

The impact of plant-derived antioxidants on the emission behavior of biodiesel blends is summarized in Table 2. In general, natural extracts exhibited lower NO_*x*_ reduction efficacy than synthetic antioxidants. At a concentration of 500 ppm, *Moringa oleifera* leaf extract, olive pomace extract, and citrus residue extract reduced NO_*x*_ emissions by 5.33%, 2.83%, and 2.39%, respectively, averaged across all four biodiesels. However, significant improvements were observed at higher concentrations (700–1000 ppm). *Moringa oleifera* extract demonstrated the highest NO_*x*_ reduction efficiency among natural antioxidants, with decreases of 16.67%, 14.97%, 15.18%, and 15.93% for HOMEs, KOMEs, COMEs, and AOMEs, respectively, at 1000 ppm. Olive pomace extract at 1000 ppm achieved NO_*x*_ reductions between 7.69% and 9.36%, while citrus residue extract produced more modest reductions ranging from 6.59% to 7.22%.

**Table 2:** Emissions from B20 blends of biodiesels enriched with natural and synthetic antioxidants

Biodiesel blends (B20)	20% HOMEs			20% KOMEs			20% COMEs			20% AOMEs		
Antioxidants concentration	CO (ppm)	NO_*x*_ (ppm)	UBHC (ppm)	CO (ppm)	NO_*x*_ (ppm)	UBHC (ppm)	CO (ppm)	NO_*x*_ (ppm)	UBHC (ppm)	CO (ppm)	NO_*x*_ (ppm)	UBHC (ppm)
B20	445	180	63	492	167	76	470	171	71	451	182	58
BHT (500 ppm)	460	168	68	502	157	78	481	160	73	463	171	71
BHT (700 ppm)	510	158	71	542	148	82	521	150	77	513	161	74
BHT (1000 ppm)	530	148	79	550	135	88	529	140	83	533	151	82
BHA (500 ppm)	455	165	65	507	158	81	486	163	76	458	168	68
BHA (700 ppm)	497	153	70	555	147	85	534	151	80	500	156	73
BHA (1000 ppm)	505	144	75	576	136	92	555	138	87	508	147	78
PG (500 ppm)	470	165	67	515	154	79	494	157	74	473	168	70
PG (700 ppm)	505	155	70	550	143	82	529	148	77	508	158	73
PG (1000 ppm)	524	143	75	571	135	89	550	137	84	527	146	78
*M. oleifera* extract (500 ppm)	450	170	60	493	158	72	476	160	67	453	173	63
*M. oleifera* (700 ppm)	455	161	63	498	149	76	480	152	71	458	164	66
*M. oleifera* (1000 ppm)	467	150	65	505	142	78	486	145	73	470	153	68
*Olive pomace* (500 ppm)	452	175	63	497	162	76	477	165	71	455	178	66
*Olive pomace* (700 ppm)	455	170	64	506	157	77	481	159	72	458	173	67
*Olive pomace* (1000 ppm)	465	165	69	517	153	82	488	155	77	468	168	72
Citrus residue extract (500 ppm)	450	175	58	495	163	71	474	169	66	453	178	61
Citrus residue extract (700 ppm)	454	172	62	511	159	76	479	165	71	457	175	65
Citrus residue extract (1000 ppm)	459	167	67	522	155	80	483	159	75	461	170	70

Regarding CO emissions, the average increase across the four biodiesels was highest for olive pomace extract (4.29%), followed by *Moringa oleifera* extract (3.80%) and citrus residue extract (3.56%). The smallest CO increase (2.22%) was recorded with citrus extract in AOMEs. For UBHC emissions, the average increment was highest with olive pomace extract (12.50%), followed by citrus extract (9.98%), while *Moringa oleifera* extract resulted in the lowest average increase (6.97%). Notably, *Moringa oleifera* extract in HOMEs led to only a 3.17% increase in UBHCs, indicating its superior stabilizing ability. Although all three natural extracts caused some elevation in CO and UBHC emissions, the magnitude of increase was considerably lower than that observed with synthetic antioxidants.

These findings highlight the promising potential of plant-derived antioxidants in emission control. The results suggest that *Moringa oleifera* extract offers the most efficient NO_*x*_ mitigation, followed by olive pomace and citrus extracts. Moreover, optimization of extraction methods (*e.g.*, employing green extraction technologies) and concentration levels could further enhance the antioxidative and emission-reducing capabilities of such bioactive compounds. The present trends are consistent with the observations of previously reported literature. For instance, Afzal *et al.* reported the significant reductions in NO_*x*_ emissions upon the addition of both synthetic and plant-based antioxidants to biodiesel fuels.^37^ Rajamohan *et al.*, reported the antioxidant potential of synthetic antioxidants (BHT, PG, TBHQ and Pyrogallol) to improve the oxidation stability for storage, combustion characteristics and engine performance of *Prosopis juliflora* biodiesel/diesel fuel blends. However, the use of synthetic antioxidants increased the emissions of CO and UBHC in the combustion studies.^40^ Jeyakumar *et al.*, reported the antioxidant potential of sesame, horse gram, sweet basil, coffee, and peas to improve the shelf life of binary biodiesel blend of Jatropha biodiesel and lemongrass oil. They reported the substantial improvement in the oxidation stability of biodiesel in the following order: sesame > horse gram > sweet basil > coffee > peas.^41^ Similarly, Jeyakumar *et al.*, also reported the improvement in the combustion properties of *Pithecellobium Dulce* seed-derived biodiesel by the addition of *Groundnut* shell nanoparticles.^42^ All these findings demonstrated the beneficial effect of natural antioxidants on the oxidation stability of the biodiesel, as reported by our findings.

## 4 Conclusion

This study underscores the crucial role of antioxidants in enhancing both the oxidative stability and emission characteristics of biodiesel derived from non-edible oils such as hemp, karanja, castor, and amla. Among the tested antioxidants, the synthetic compound BHA exhibited the most effective stabilization performance, showing the least increase in oxidation parameters. Among the natural antioxidants, *Moringa oleifera* leaf extract and olive pomace extract proved to be the most efficient, with *M. oleifera* offering superior control over conjugated dienes and trienes, while olive pomace extract effectively stabilized peroxide and totox values. Overall, karanja oil biodiesel demonstrated the highest oxidative stability after 28 days of storage. In emission analyses, the inclusion of synthetic antioxidants markedly reduced NO_*x*_ emissions, following the order of effectiveness PG > BHA > BHT. However, this reduction was accompanied by a corresponding rise in CO and unburnt hydrocarbon (UBHC) emissions, increasing in the order BHA < PG < BHT. Natural antioxidants showed moderate NO_*x*_ reduction potential but exerted less influence on CO and UBHC emissions. Among the plant extracts, citrus residue extract was most effective in limiting CO emissions, while *Moringa oleifera* extract exhibited the greatest reduction in UBHC and NO_*x*_ emissions, followed by olive pomace extract. Overall, the findings confirm that both synthetic and natural antioxidants can significantly enhance biodiesel stability and emission performance. The use of natural, locally available plant extracts offers a promising and sustainable strategy for improving biodiesel quality while mitigating environmental impacts associated with fossil fuel combustion.

## Author contributions

Humaira Kanwal: Conceptualization, methodology, investigation, writing original draft. Farooq Anwar: Supervision, funding acquisition, writing review & editing. Ahsan Tanvir: Formal analysis, writing original draft, review & editing. Abu Bakar Siddique: Formal analysis, writing review & editing. Sobia Tariq: Data curation, visualization. Sidra Aslam: investigation, review & editing. Saiqa Yaqoob: investigation, writing.

## Conflicts of interest

The authors declare no conflicts of interest related to this research.

## Data Availability

All evaluated data is available in the manuscript. Additional information/data can be provided upon reasonable request.
